# Stromally Expressed β-Catenin Modulates Wnt9b Signaling in the Ureteric Epithelium

**DOI:** 10.1371/journal.pone.0120347

**Published:** 2015-03-24

**Authors:** Felix J. Boivin, Sanjay Sarin, Janice Lim, Ashkan Javidan, Bruno Svajger, Hadiseh Khalili, Darren Bridgewater

**Affiliations:** Department of Pathology and Molecular Medicine, McMaster University, Hamilton, Canada; UCL Institute of Child Health, UNITED KINGDOM

## Abstract

The mammalian kidney undergoes cell interactions between the epithelium and mesenchyme to form the essential filtration unit of the kidney, termed the nephron. A third cell type, the kidney stroma, is a population of fibroblasts located in the kidney capsule, cortex and medulla and is ideally located to affect kidney formation. We found β-catenin, a transcriptional co-activator, is strongly expressed in distinctive intracellular patterns in the capsular, cortical, and medullary renal stroma. We investigated β-catenin function in the renal stroma using a conditional knockout strategy that genetically deleted β-catenin specifically in the renal stroma cell lineage (β-cat^s-/-^). *β-cat^s-/-^* mutant mice demonstrate marked kidney abnormalities, and surprisingly we show β-catenin in the renal stroma is essential for regulating the condensing mesenchyme cell population. We show that the population of induced mesenchyme cells is significantly reduced in *β-cat^s-/-^* mutants and exhibited decreased cell proliferation and a specific loss of Cited 1, while maintaining the expression of other essential nephron progenitor proteins. *Wnt9b*, the key signal for the induction of nephron progenitors, was markedly reduced in adjacent ureteric epithelial cells in *β-cat^s-/-^*. Analysis of Wnt9b-dependent genes in the neighboring nephron progenitors was significantly reduced while Wnt9b-independent genes remained unchanged. In contrast mice overexpressing β-catenin exclusively in the renal stroma demonstrated massive increases in the condensing mesenchyme population and *Wnt9b* was markedly elevated. We propose that β-catenin in the renal stroma modulates a genetic program in ureteric epithelium that is required for the induction of nephron progenitors.

## Introduction

Development of the mammalian kidney is dependent upon inductive interactions between epithelial and mesenchymal cells [1]. Development of the mature kidney is initiated with the outgrowth of an epithelial tube, termed the ureteric bud, which elongates and migrates into a population of mesenchyme, termed the metanephric mesenchyme (MM) beginning at embryonic (E) day 10.5. The ureteric cells send signals to the adjacent mesenchyme that instruct the MM to condense around the tips of the ureteric epithelium forming a population of induced nephron progenitors. These nephron progenitors undergo a mesenchymal-to-epithelial transition and progress through a series of molecular and morphological changes to form the nephron, the filtering units of the kidney. In reciprocal fashion the MM cells signal to the ureteric epithelial cells to promote growth and reiterative branching of the ureteric epithelial cells to form the collecting system of the kidney [2], [3]. By E11.5, two distinct cell populations are established within the MM, the nephrogenic progenitors and the renal stroma. The reciprocal signaling interactions between the nephrogenic progenitors and the ureteric epithelium during kidney development are well characterized. However, the role of the renal stroma and its interactions with the nephrogenic progenitors and epithelium are poorly understood during kidney development.

The renal stroma is a population of matrix-producing fibroblast cells [4], [5]. During kidney development the stromal population is initially observed after the ureteric epithelium invades the MM, as evidenced by the expression of the earliest stromal differentiation marker Foxd1 [4]. At the initiation of kidney development, the Foxd1 positive cells are loosely packed cells positioned adjacent to the condensing mesenchyme [4]. As the ureteric epithelium continues to branch, the stromal cells are positioned around the maturing epithelium and newly forming nephrons. As the kidney develops beyond E14.5, the renal stroma subdivides into three distinct cell populations: the capsular, cortical and medullary stroma. The function of the renal stroma was originally thought to provide a supportive framework for the nephrons and collecting duct system [6]. However, deletion of Foxd1 in the developing kidney results in defects in branching morphogenesis and nephrogenesis, suggesting an essential role for the renal stroma in kidney development [4]. Recent studies demonstrated that a complete ablation of the renal stromal resulted in increased induced nephron progenitors and significantly disrupted branching morphogenesis [7], [8]. Moreover, the renal stroma controls nephron differentiation by forming direct cell interactions with the nephrogenic progenitors [7]. These studies highlight the importance of reciprocal interactions between stroma, mesenchyme, and epithelium. However the stromal factors that guide these interactions are poorly defined.

β-catenin is a multi-functional protein involved in cell adhesion and the regulation of gene transcription. In the cell, β-catenin localizes to the cell membrane, cytoplasm, and nucleus where it performs different cellular functions. At the cell membrane β-catenin is involved in the formation, maintenance, and function of adherens junctions by linking cadherins to the actin cytoskeleton [9]. In the cytoplasm, β-catenin is involved in cell signal transduction by translocating to the nucleus in response to extracellular stimuli. In the nucleus β-catenin acts as a co-transcriptional activator by forming complexes with DNA bound transcription factors to regulate gene transcription [10]. During kidney development, β-catenin is expressed in the ureteric epithelium and plays essential roles in branching morphogenesis and in the differentiation of ureteric epithelial cells through the regulation of key genetic targets [11], [12]. β-catenin expression in the mesenchyme is essential for nephron formation by mediating signals necessary for the induction of nephron progenitors [13]. Previously a role for β-catenin mediated signaling was demonstrated in the medullary stroma. In response to Wnt7b, secreted by ureteric epithelial cells, the medullary stroma activates a β-catenin-mediated canonical signaling pathway to control proper patterning of the cortico-medullary axis and elongation of epithelial structures [14]. However, the expression of β-catenin in stromal cells and the molecular mechanism by which stromally expressed β-catenin controls kidney development in not known.

In this study, we investigated the role of stromally expressed β-catenin in kidney development. We show β-catenin expression in distinctive intracellular patterns in the capsular, cortical, and medullary renal stroma. To understand the significance of stromally expressed β-catenin we generated a conditional knockout mouse in which β-catenin is specifically deleted in the renal stroma cell lineage (*β-cat* ^*s-/*-^). These mutant mice demonstrate marked kidney abnormalities, most notably reductions in the condensing mesenchyme. Using in situ hybridization and real-time quantitative PCR (qRT-PCR), we demonstrated *Wnt9b*, the key signal for the induction of nephron progenitors, was markedly reduced in ureteric epithelial cells in *β-cat* ^*s-/*-^. Consistent with reduced levels of *Wnt9b*, we demonstrated a significant down regulation of Wnt9b-dependent genes, while Wnt9b-independent genes remained unchanged in nephron progenitors. Moreover mice overexpressing β-catenin exclusively in the renal stroma demonstrated massive increases in induced nephron progenitors and *Wnt9b*. Together these data support a model in which β-catenin in the renal stroma communicates with the ureteric epithelium to modulate a genetic program that is required for the induction of nephron progenitors during mammalian kidney development.

## Results / Discussion

### β-catenin is expressed in distinctive patterns in the renal stroma

**Fig 1:**
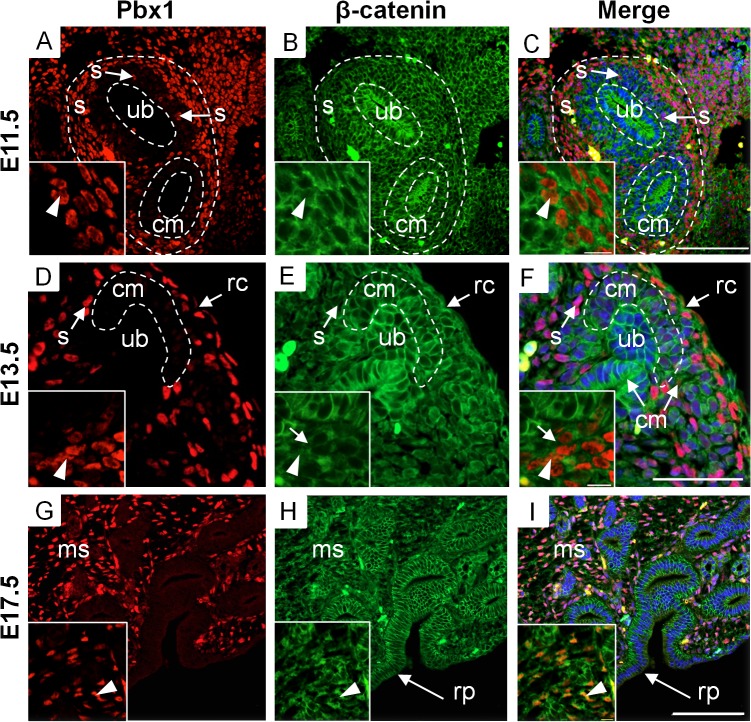
β-catenin is expressed in distinctive patterns in the renal stroma. (A-I) Immunofluorescence demonstrating β-catenin spatial and temporal expression in stromal cells. (A) At E11.5 Pbx1 is expressed in the nucleus of stromal cells (arrow head-inset) surrounding the condensing mesenchyme. Some Pbx1 positive stromal cells locate within the condensing mesenchyme, directly adjacent to epithelial cells (arrows). (B,C) At E11.5 β-catenin is expressed in the condensing mesenchyme and ureteric epithelium, and co-localizes with Pbx1 demonstrating expression in the renal stroma. At E11.5, some Pbx1 cells co-localize with β-catenin in the nuclear compartment of stromal cells (arrowhead-inset). (D) At E13.5, Pbx1 is expressed in capsular and cortical stroma surrounding the condensing mesenchyme. (E, F) At E13.5, β-catenin co-localizes in the cytoplasm of capsular stromal cells. The stromal cells located between developing nephrons express β-catenin in the cytoplasmic (arrow-inset) and nuclear compartment (arrowhead-inset). (G) At E17.5, Pbx1 marks the capsular, cortical, and medullary stroma. (H-I) β-catenin is expressed in the medullary stroma and co-localizes strongly with Pbx1 in the nuclear compartment (arrowhead-inset). (scale bar = 100μm, s = stroma, cm = condensing mesenchyme, ub = ureteric epithelium, rc = renal capsule, ms = medullary stroma, rp = renal pelvis).

To establish the expression pattern of β-catenin in the renal stroma we performed co-immunofluorescence using antibodies to β-catenin and the renal stromal nuclear marker Pbx1. We utilized a β-catenin antibody that recognizes all forms of the β-catenin protein (cadherin-bound, active, and inactive), and localizes to the membrane, cytoplasm and nucleus [15]. The renal stroma is initially observed at E11.5 after the ureteric epithelium invades the mesenchyme [4]. At E11.5 Pbx1, a marker of all stromal cells (S1 Fig.), is expressed in stromal cells surrounding the condensed mesenchyme and is primarily absent from the condensed mesenchyme itself (Fig. 1A). We confirmed our previous findings that β-catenin is expressed in the condensed mesenchyme and ureteric epithelium [11] (Fig. 1B) and here we further demonstrate β-catenin co-localizes with stromal marker Pbx1 (Fig. 1C). At E11.5, the intracellular distribution of β-catenin in Pbx1 positive cells is primarily cytoplasmic, and virtually absent from the nucleus (Fig. 1B and C inset). Interestingly, some Pbx1 positive cells are found within the condensed mesenchyme population in close proximity to the ureteric epithelium (Fig. 1A and C - arrow). Stromal-epithelial interactions have been shown to be essential in prostate and mammary branching morphogenesis [16], [17]. Therefore, the close proximity between the stroma and the ureteric epithelium may suggest possible cell-cell interactions between the renal stroma and ureteric epithelial cells.

At E13.5, the Pbx1 positive renal stroma cells are located between adjacent condensed mesenchymal populations and also form the renal capsule [18] (Fig. 1D). β-catenin is expressed in both the capsular stromal cells and stromal cells located between the condensed mesenchyme (Fig. 1 E, F). The cortical-medullary axis is established at E14.5, and after this time point [19] the renal stroma is divided into the capsular, cortical, and medullary stroma. We observed β-catenin expression at E17.5 in cortical and capsular stroma in a pattern identical to that observed at E13.5. Further at E17.5 β-catenin is expressed in the medullary stroma (Fig. 1G-I). Our temporal analysis reveals that β-catenin is expressed at the onset of stromal cell formation and is maintained in the different stromal cell populations throughout kidney development.

**Fig 2:**
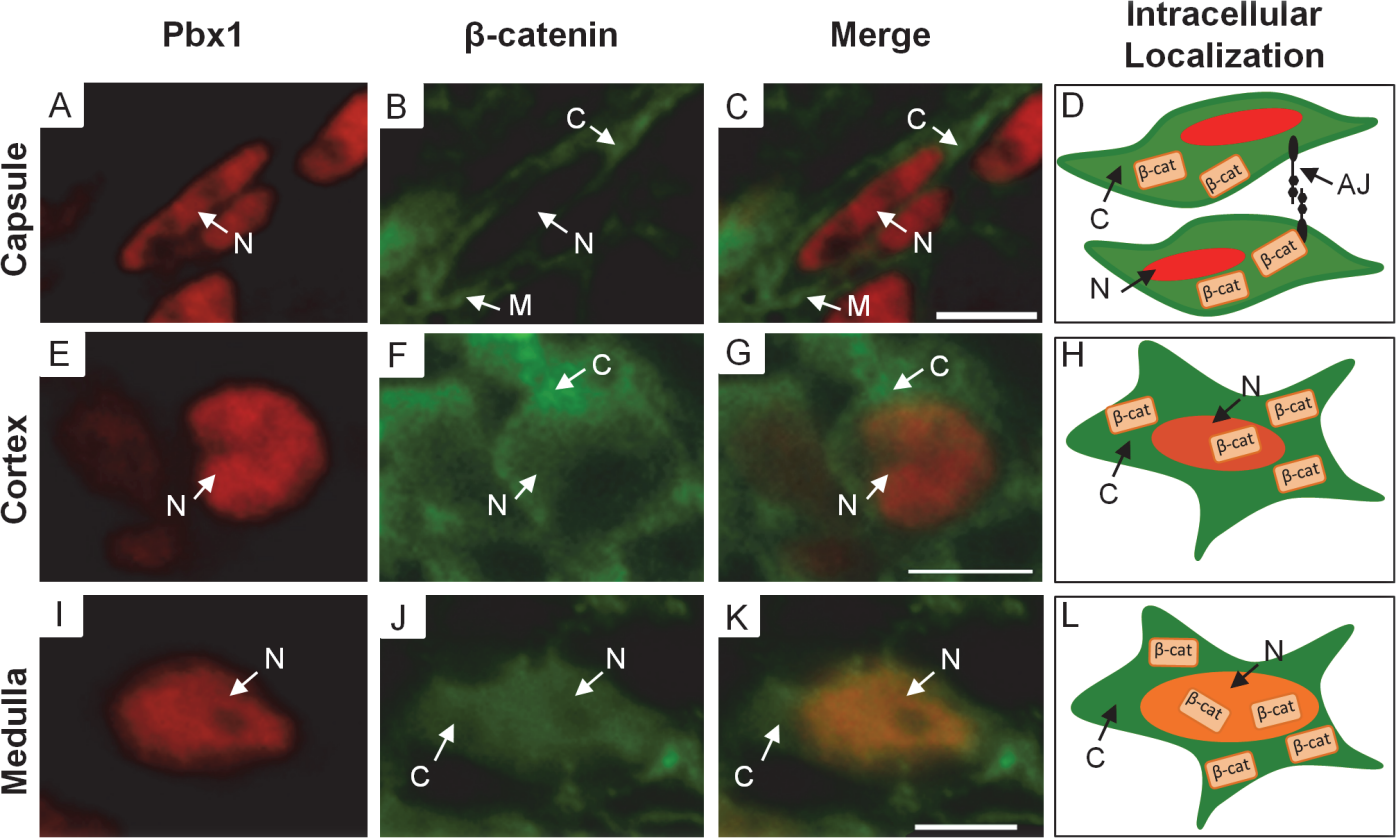
Intracellular localization of β-catenin in capsular, cortical, and medullary stroma. (A-L) Immunofluorescence showing β-catenin intracellular distribution in the capsular, cortical, and medullary stroma. (A-C) In the capsular stroma, β-catenin localizes in a membrane and cytoplasmic pattern and does not co-localize with nuclear stromal factor Pbx1. (D) Schematic diagram of the intracellular β-catenin localization and a suggested role in cell-cell adhesion via adherens junctions. (E-G) β-catenin weakly co-localizes with Pbx1 in the nuclear compartment but is primarily cytoplasmic. (H) Schematic diagram of the intracellular β-catenin localization showing possible roles in the cytoplasm and nucleus. (I-K) In the medulla, β-catenin co-localizes with Pbx1 primarily to the nuclear compartment but some cytoplasmic β-catenin expression is observed. (L) Schematic diagram of the intracellular β-catenin localization showing a more prominent role in the nucleus. (scale bar = 5μm, M = membrane, C = cytoplasm, N = nuclear, AJ = Adherens junctions).

During our analysis of β-catenin expression, we observed that the intracellular distribution of β-catenin varied between the capsular, cortical, and medullary stromal compartments. In the capsular stroma, the intracellular distribution of β-catenin is primarily in the cytoplasm and membrane and virtually absent from the nucleus (Fig. 2A-D), indicating a more prominent role in cell adhesion. In the population of cortical stroma, β-catenin is cytoplasmic and low levels are observed in the nucleus (Fig. 2E-H). This intracellular pattern suggests an important role in cell adhesion with a more minor role in signaling and gene transcription. In contrast, within the medullary stroma, β-catenin is most highly expressed in the nuclei with lower levels in the cytoplasm (Fig. 2I-L), suggesting a prominent role in the regulation of gene transcription. This intracellular distribution of β-catenin within these stromal compartments was maintained throughout kidney development. These results demonstrate that each stromal cell population has a unique intracellular distribution of β-catenin suggesting unique functional roles during kidney development.

### Ablation of β-catenin in stromal cells leads to multiply kidney abnormalities

To investigate the role of stromally expressed β-catenin in kidney development we generated a conditional knockout mouse model whereby β-catenin was specifically deleted in the renal stroma cell lineage. We utilized a *Foxd1-Cre* transgenic mouse line [20] that expresses the Cre recombinase protein exclusively in the renal stroma [7], [8]. We crossed the *Foxd1-Cre* mice with transgenic mice containing *LoxP* sites flanking exons 2–6 of the *β-catenin* allele [21] resulting in the generation of mice with a genetic deletion of β-catenin from renal stromal cells (termed *β-cat* ^*S-/*-^). We performed immunofluorescence using Pbx1 and β-catenin antibodies to confirm the absence of β-catenin within all stromal cells in *β-cat* ^*S-/*-^ mutant kidneys (S2 Fig.). Our results demonstrate β-catenin expression was maintained in the condensed mesenchyme and ureteric epithelium but completely absent in Pbx1 positive cells during embryonic kidney development and at post-natal day 0 (PN0) in the cortex and medulla (S2 Fig.).

**Fig 3:**
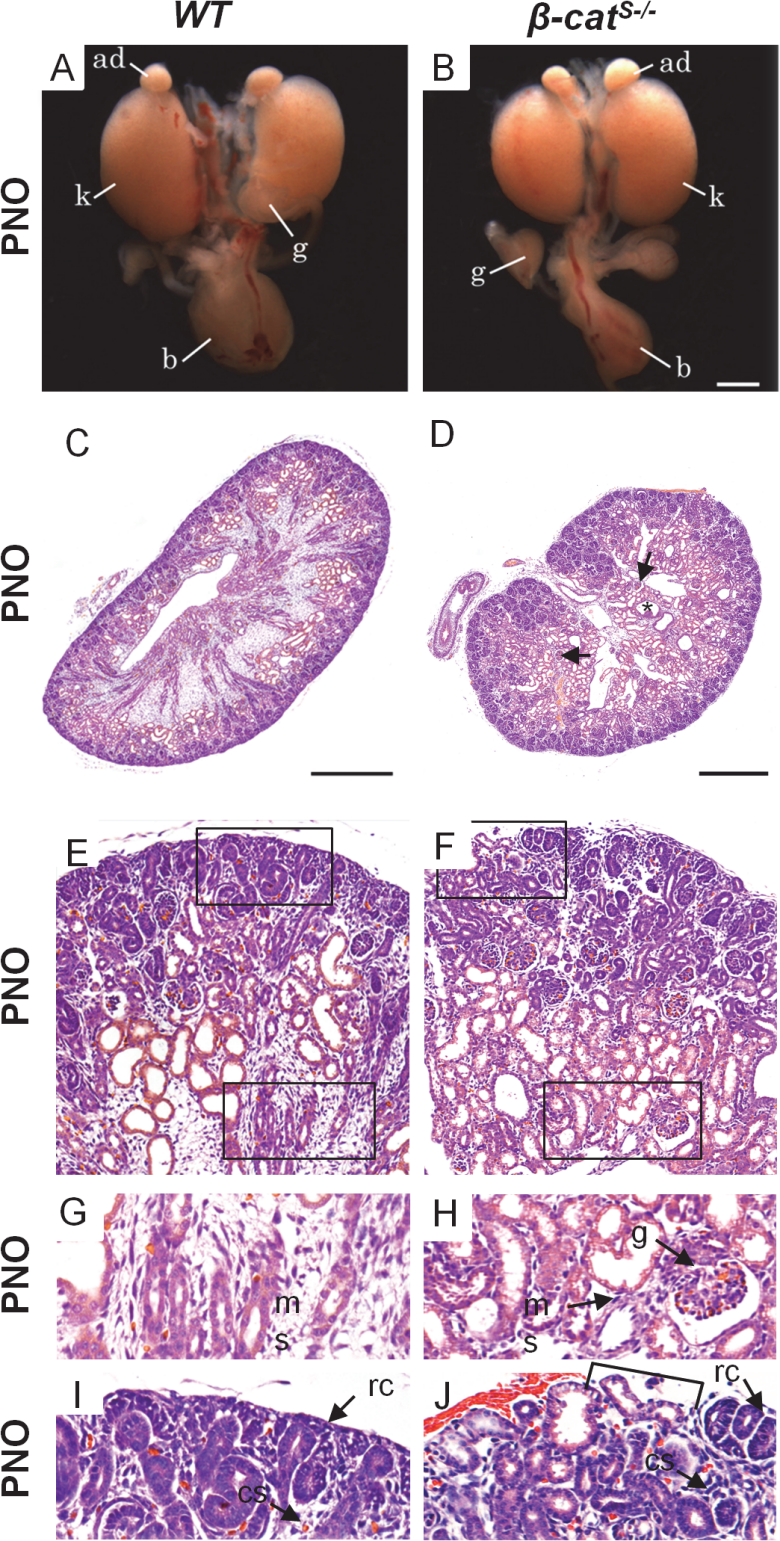
*β-cat* ^*S-/*-^ mutants demonstrate multiple kidney abnormalities. (A,B) Gross anatomy of PN0 *WT* and *β-cat* ^*S-/*-^ kidneys show comparable size and shape. (C-J) In contrast to *WT*, histological analysis of *β-cat* ^*S-/*-^ mutant kidneys demonstrate numerous kidney abnormalities. (C,D) As compared to *WT*, *β-cat* ^*S-/*-^ mutant kidneys were lobular, lacked a distinct boarder, contained numerous cysts in the medulla and cortex (star), with an ill-defined cortical medullary axis and misplaced glomeruli (arrow). (E-J) In contrast to *WT*, high magnification of *β-cat* ^*S-/*-^ kidneys at PN0 revealed a non-adherent sporadic renal capsule (F and J), misplaced tubules just under the renal capsule (F and J), glomeruli in the medulla (H) and a marked reduction in medullary stroma (H). (A, B scale bar = 1mm, C, D scale bar = 100μm, ad = adrenal gland, k = kidney, b = bladder, rc = renal capsule, cs = cortical stroma, ms = medullary stroma, g = glomerulus).

*β-cat* ^*S-/*-^ mice died within hours after birth and kidney tissue was immediately isolated for gross and histological analysis. Analysis of the gross anatomy of kidneys from 7 different *β-cat* ^*S-/*-^ mutants at PN0 revealed 6 mutants with normal to slightly smaller kidneys when compared to wild-type (*WT*) (Fig. 3A and B). One mutant demonstrated kidneys that were 50% smaller than *WT* littermates (data not shown). We then performed a histological analysis of *β-cat* ^*S-/*-^ kidneys. In contrast to *WT* (Fig. 3C), *β-cat* ^*S-/*-^ mutant kidneys were lobular and contained numerous large ureteric epithelial derived cysts (S3 Fig.) in the medulla and cortex (Fig. 3D). In addition, we observed glomeruli abnormally located in the medulla (Fig. 3 E-H) and tubules were inappropriately located just beneath the renal capsule (Fig. 3E, F, I, J). *β-cat* ^*S-/*-^ mutant kidneys demonstrate a phenotype that is consistent with previous reports by Yu et al [14]. Yu et al demonstrated that the loss of β-catenin from the medullary stroma cells resulted in the cystic transformation of collecting ducts [14]. Their study concluded that ureteric epithelial cells secrete Wnt7b which activates the canonical Wnt pathway in medullary stroma resulting in elongation and maintenance of loops of Henle and collecting ducts [14].

In addition, *β-cat* ^*S-/*-^ kidneys revealed a paucity of renal capsule and sporadic non-adherent capsular cells (Fig. 3 I,J). This data combined with our expression analysis in which β-catenin primarily localizes to the cell membrane of the capsular stroma suggest a primary role in cell-cell adhesion. However, there are regions where the renal capsule remains intact and this could be due to the compensatory role of γ-catenin (plakoglobin) as previously described [22].

**Fig 4:**
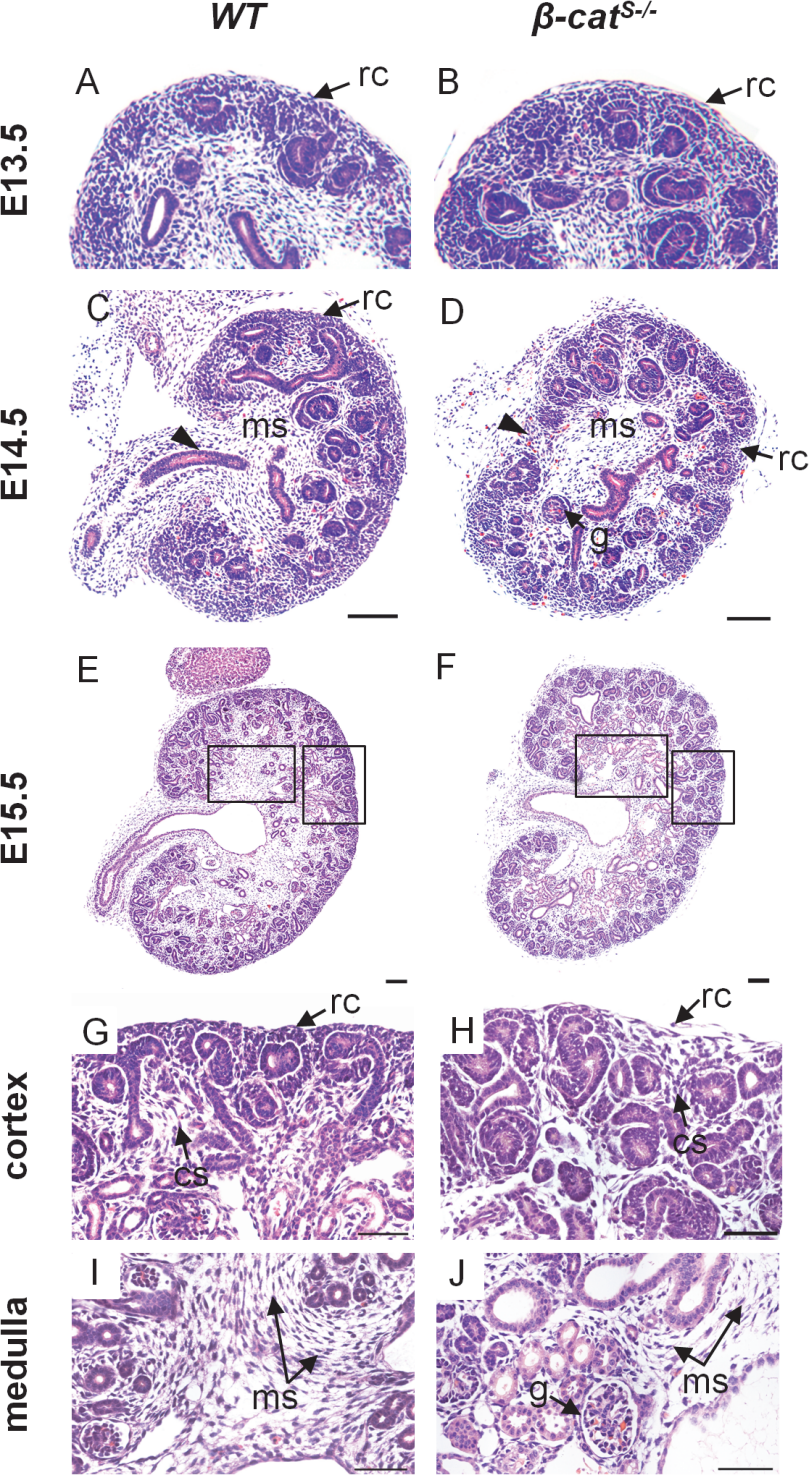
Temporal analysis of the embryonic kidney phenotype in *β-cat* ^*S-/*-^ mutants. (A, B) Histological analysis of *WT* and *β-cat* ^*S-/*-^ embryonic kidneys at E13.5 demonstrates no abnormalities in the stromal population, developing nephrons, or kidney patterning. (C, D) In contrast to *WT* at E14.5, *β-cat* ^*S-/*-^ kidneys demonstrate abnormally located glomeruli, and a non-adherent irregular patterned renal capsule. (E-J) In contrast to *WT* at E15.5, the non-adherent capsular phenotype persists in *β-cat* ^*S-/*-^ kidneys (H) and the cortical stroma is reduced and loosely packed (H). Similarly, the medullary stroma in *β-cat* ^*S-/*-^ kidneys is markedly reduced compared to *WT* (J) and glomeruli are also abnormally located within the medulla (rc = renal capsule, cs = cortical stroma, ms = medullary stroma, g = glomerulus, arrowhead = ureter). Scale Bar C-F = 100μm, G-J = 50μm

**Fig 5:**
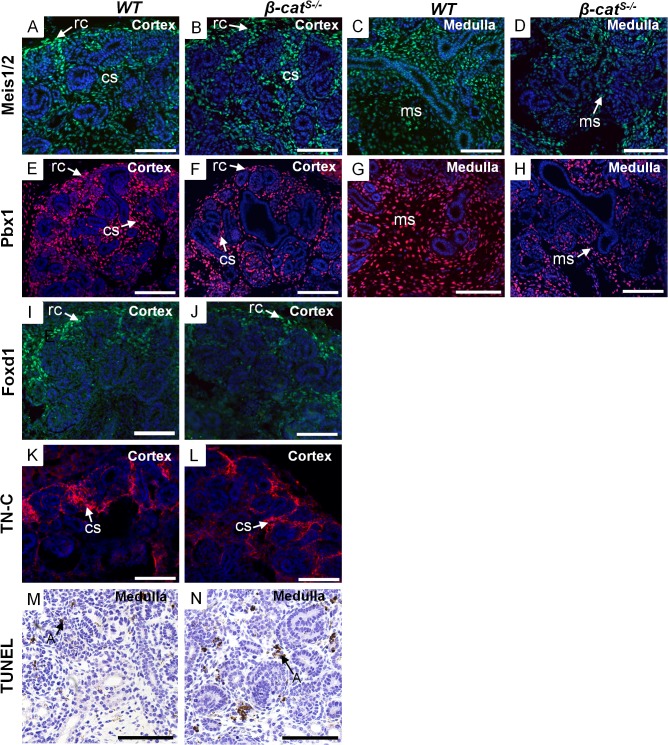
Investigation of the renal stroma in *β-cat* ^*S-/*-^ mutant kidneys. (A-L) Analysis of the renal stroma using stromal markers; Meis1/2, Pbx1, Foxd1, and TN-C at E15.5. As compared to *WT*, no overt changes were observed in the cortical stroma with respect to Meis1/2 (A, B), Pbx1 (E, F), Foxd1 (I, J), and TN-C (K, L). However, a reduction of Meis1/2 (C, D) and Pbx1 (G, H) was observed in the medullary region in *β-cat* ^*S-/*-^ kidneys. (M, N) TUNEL assay at E15.5 reveals an increase in apoptosis in the medullary stroma of *β-cat* ^*S-/*-^ kidneys compared to *WT*. (rc = renal capsule, cs = cortical stroma, ms = medullary stroma, A = apoptosis). Scale Bar = 50μm

To determine when the mutant phenotype is initially established, we performed a histological analysis of *β-cat* ^*S-/*-^ kidneys at various embryonic time points including E13.5, E14.5, and E15.5. *β-cat* ^*S-/*-^ kidneys at E13.5 were indistinguishable from *WT* littermates (Fig. 4A, B). By E14.5, *β-cat* ^*S-/*-^ kidneys revealed glomeruli in the medulla and a loosely adherent renal capsule (Fig. 4C, D). At E15.5, *β-cat* ^*S-/*-^ kidneys displayed sparse, loosely packed cortical stroma between the adjacent condensed mesenchymal populations, glomeruli in the medulla, sparse medullary stroma, and the non-adherent renal capsule phenotype persisted (Fig. 4E-J). We also analyzed branching morphogenesis and nephrogenesis and observed no overt changes in branch generation or nephrogenesis in *β-cat* ^*S-/*-^ kidneys (S4 Fig.). We next analyzed the renal stroma cell population using various stromal markers Meis1/2, Foxd1, Tenascin C, and Pbx1 (Fig. 5A-L). While the capsular, cortical, and medullary stroma were present we noted a reduction in the medullary stromal cell population (Fig. 5C,D,G,H). To determine if apoptosis was a contributing factor to the reduced medullary stroma we performed a TUNEL analysis. Indeed the analysis showed increases in apoptosis within the medullary stroma at E15.5 (Fig. 5M,N). Combined, these studies highlight the role of stromally expressed β-catenin in the proper formation of the distinct stromal compartments.

### Stromally expressed β-catenin modulates Wnt9b expression in the ureteric epithelium

**Fig 6:**
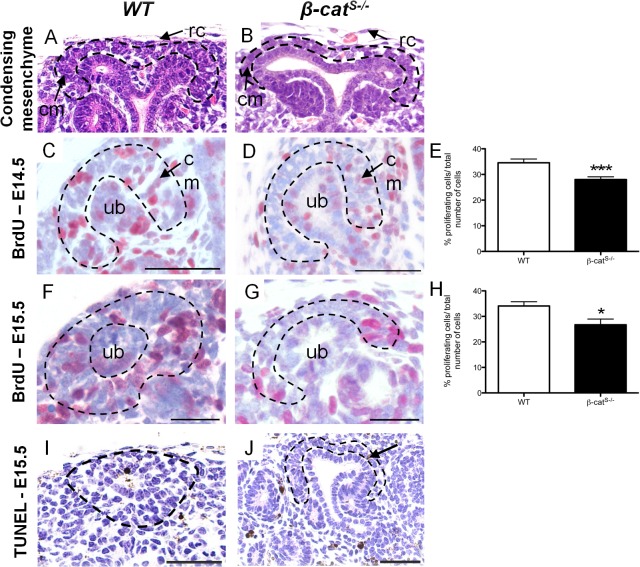
The condensing mesenchyme cell population is reduced in *β-cat* ^*S-/*-^ mutant kidneys. (A,B) As compared to *WT*, which demonstrates 3–4 cell layers of aggregated condensing mesenchyme, *β-cat* ^*S-/*-^ kidneys display a reduced, single cell layer of loosely aggregated condensing mesenchyme. (C-H) Analysis of cell proliferation in the condensing mesenchyme was performed using Brdu cell proliferation assay. (C-E) As compared to *WT*, *β-cat* ^*S-/*-^ mutants demonstrated a 6.46% reduction in condensing mesenchyme cell proliferation at E14.5 (*WT*, 34.56%±1.45, n = 28 versus *β-cat* ^*S-/*-^, 28.02%±1.05, n = 27, p = 0.0006). (F-G) At E15.5 *β-cat* ^*S-/*-^ mutants demonstrated a 7.35% reduction in condensing mesenchyme cell proliferation when compared to *WT* (*WT*, 34.09%±1.65, n = 17 versus *β-cat* ^*S-/*-^, 26.73%±2.21, n = 15, p = 0.01). (I,J) A TUNEL assay at E15.5 did not reveal any changes in apoptosis in the condensing mesenchyme between *WT* and *β-cat* ^*S-/*-^. Scale Bar = 50μm

During kidney development the ureteric epithelial tips induce the mesenchyme population to tightly cluster around the ureteric epithelium tips and form a 3–4 cell-layer thick population of condensed mesenchymal cells [2]. Notably, the condensed mesenchyme population surrounding the ureteric epithelial tips in *β-cat* ^*S-/*-^ mutant kidneys was reduced to a single loosely packed layer of condensed mesenchymal cells when compared to *WT* (Fig. 6 A, B). This finding suggests an important role for stromally expressed β-catenin in the induction of mesenchyme progenitors during kidney development. Signals from the ureteric epithelium promote proliferation of these mesenchyme progenitors [23], [24]. We next examined whether the reduction in the condensed mesenchymal population in *β-cat* ^*S-/*-^ kidneys resulted from changes in cell proliferation. We quantified the percentage of proliferating cells at E14.5 and E15.5 specifically in the condensed mesenchyme using BrdU labeling. The percentage of proliferating condensing mesenchyme cells was reduced in *β-cat* ^*S-/*-^ mutant kidneys at E14.5 (WT: 34.56% versus *β-cat* ^*S-/*-^: *28*.*02%)* and at E15.5 (WT: 34.09% versus *β-cat* ^*S-/*-^: *26*.*73%)* (Fig. 6C-H). In contrast, we observed virtually no apoptosis within the condensing mesenchyme population in either *β-cat* ^*S-/*-^ mutants or *WT* littermates (Fig. 6I,J). This data demonstrates the reduction in the condensed mesenchyme in *β-cat* ^*S-/*-^ is most likely caused by reduced proliferation of the progenitor cell population.

**Fig 7:**
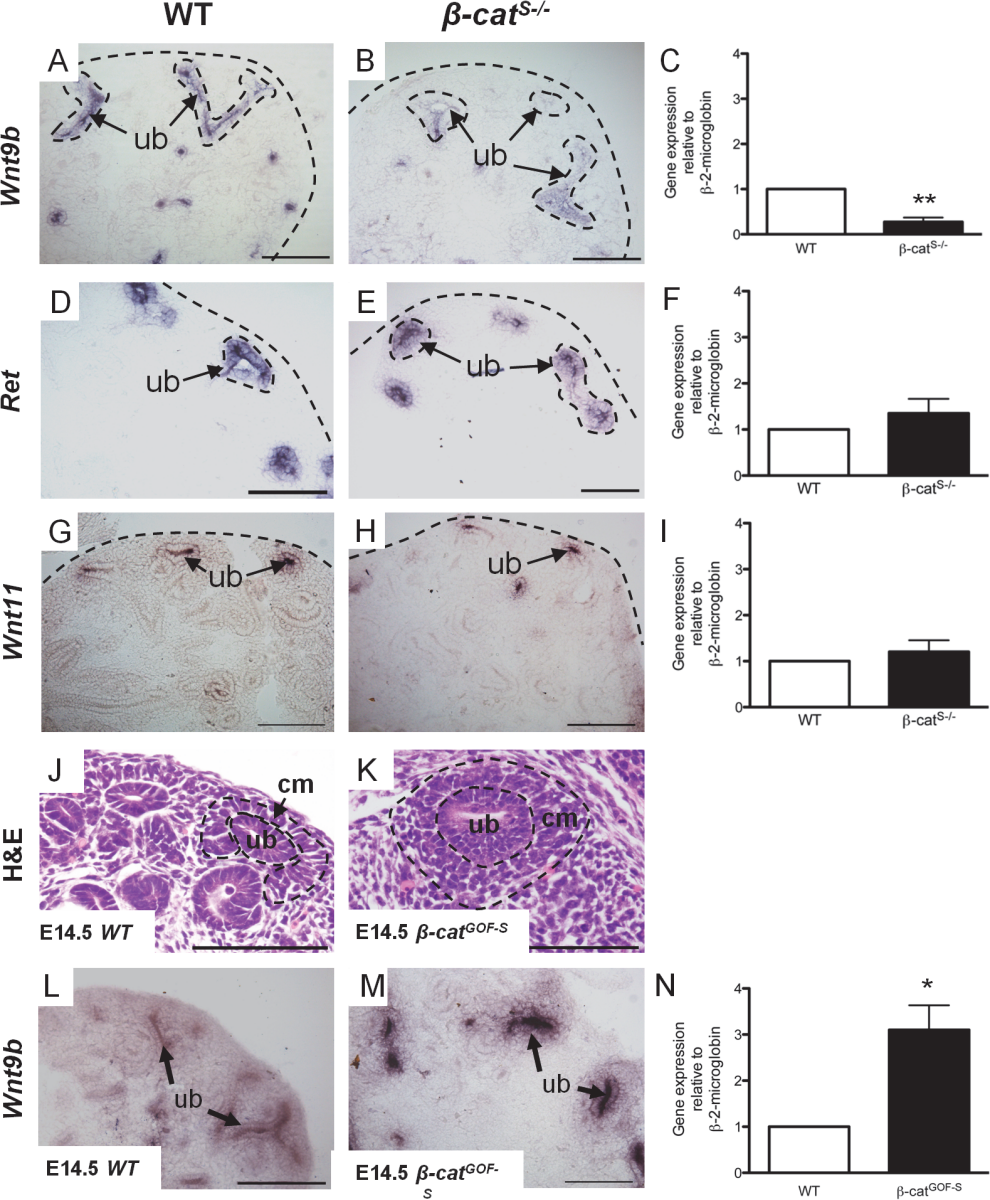
β-catenin in the renal stroma modulates Wnt9b expression in ureteric epithelial cells. (A-C) When compared to *WT*, In situ hybridization and real-time quantitative PCR for *Wnt9b* demonstrates *Wnt9b* mRNA expression is significantly reduced (1.00 versus 0.29, p=0.008) in E14.5 *β-cat^S-/-^* kidneys. (D-I) In situ hybridization and Real-time quantitative PCR for *Ret* and *Wnt11* demonstrated no differences in mRNA expression between *WT* and *β-cat^S-/-^* at E14.5. (J-K) Histological analysis of *β-cat^GOF-S^* mutant kidneys demonstrate a marked increase in condensing mesenchyme population when compared to *WT* at E14.5. (L-N) In situ hybridization and quantitative PCR demonstrate *Wnt9b* expression in *β-cat^GOF-S^* kidneys was significantly increased (1.02 versus 3.102, p=0.021) as compared to *WT* at E14.5. (scale bar = 50 μm, rc-renal capsule, cm= condensing mesenchyme, ub = ureteric epithelium).

Wnt9b is the key signaling factor secreted by the ureteric epithelium and controls the proliferation of progenitors and the induction of mesenchymal-to-epithelial transition of the nephrogenic progenitor population [24]. Therefore we analyzed *Wnt9b* expression by section in situ hybridization. We observed a marked reduction in *Wnt9b* mRNA expression in the majority of ureteric epithelial cells in *β-cat* ^*S-/*-^ kidneys (Fig. 7A,B). We performed qRT-PCR in *β-cat* ^*S-/*-^ kidneys using 5 mutant kidneys from 3 separate litters, and confirmed a 72% reduction in *Wnt9b* mRNA levels in *β-cat* ^*S-/*-^ kidneys (Fig. 7C). In support of these findings Wnt9b hypomorphs display a similar phenotype to our *β-cat* ^*S-/*-^ mutants [7], [25]. These results demonstrate a loss of β-catenin in stromal cells leads to reduced *Wnt9b* levels in the ureteric epithelium. To ensure the reductions in *Wnt9b* expression were not caused by a general disruption in the ureteric epithelium integrity, we analyzed *Ret* and *Wnt 11*, two other essential ureteric epithelial markers, and show no changes in their mRNA expression by in situ hybridization and qRT-PCR (Fig. 7D-I). These results led us to hypothesize that stromally expressed β-catenin modulates *Wnt9b* expression in the ureteric epithelium. To support this hypothesis, we generated a second mutant mouse model in which β-catenin was overexpressed exclusively in kidney stromal cells (*β-cat* ^*GOF-S*^). The histological analysis of *β-cat* ^*GOF-S*^ kidneys revealed a marked increase in the progenitor population at E14.5 (Fig. 7J,K). We next performed in situ hybridization and observed marked increases in *Wnt9b* expression at E14.5 (Fig. 7L,M). We confirmed these changes by qRT-PCR and demonstrated a 310% increase in *Wnt9b* expression in *β-cat* ^*GOF-S*^ (Fig. 7N). Taken together, analysis of *β-cat* ^*S-/*-^ and *β-cat* ^*GOF-S*^ mutants implicate stromally expressed β-catenin in the modulation of *Wnt9b* expression in the ureteric epithelium.

### Deletion of β-catenin in stromal cells impairs Wnt9b signaling to the nephrogenic progenitors

**Fig 8:**
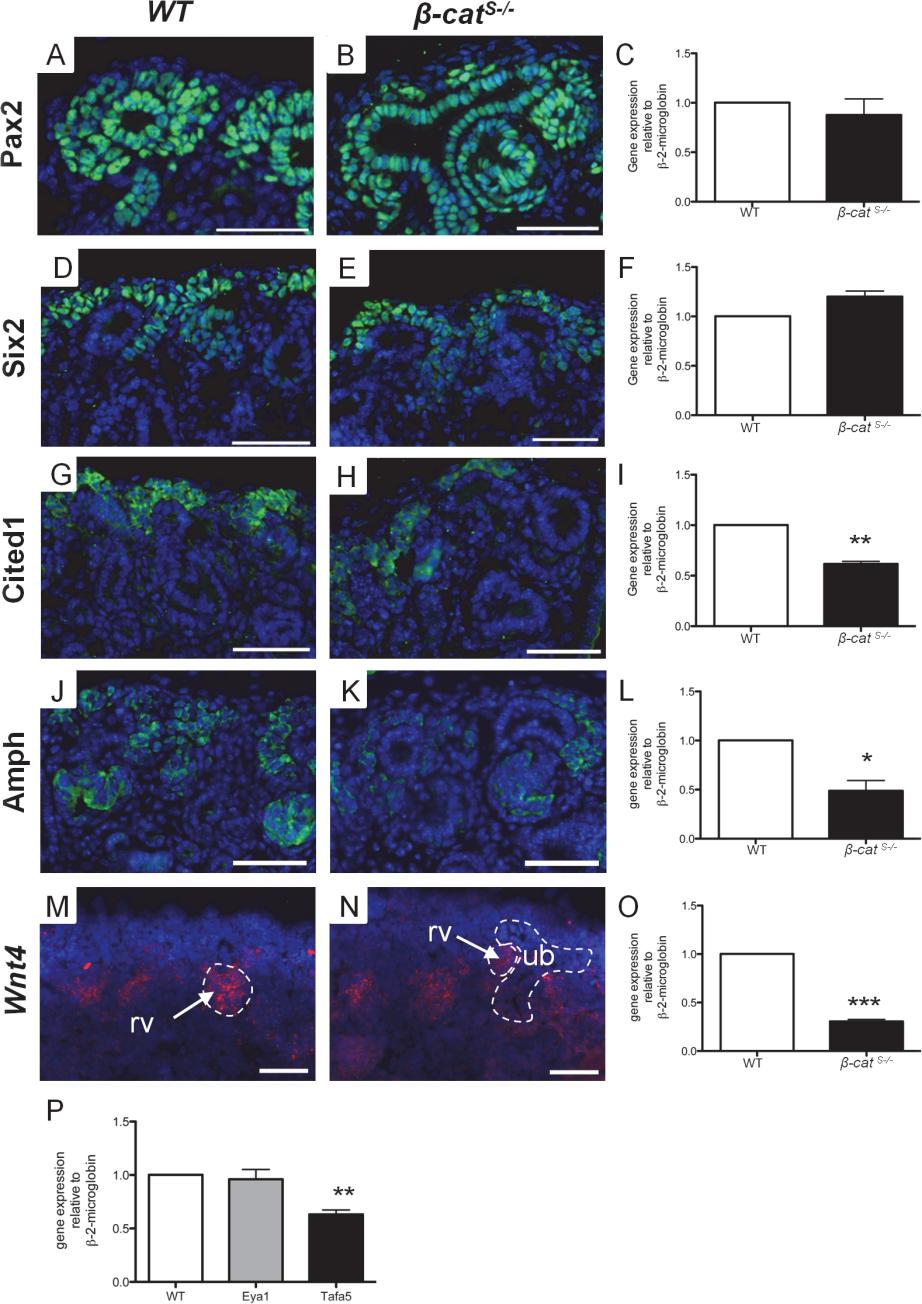
*β-cat* ^*S-/*-^ mutants demonstrate altered Wnt9b signaling to the condensing mesenchyme. (A-P) Analysis of Wnt9b dependent and independent gene targets by immunofluorescence and real-time quantitative PCR at E15.5 and E14.5 respectively. (A-F) In contrast to *WT* at E15.5, the number of Pax2 and Six2 positive cells in the condensing mesenchyme was reduced in *β-cat* ^*S-/*-^ kidneys. No changes were observed in the *Pax2* and *Six2* mRNA expression levels at E14.5 by qRT-PCR. (G-I) Both the number of Cited 1 positive cells and *Cited 1* mRNA expression levels were reduced (1.00 versus 0.62, p = 0.003) in *β-cat* ^*S-/*-^ kidneys. (J-L) The levels of Amphiphysin were significantly reduced in the condensing mesenchyme at both the protein and mRNA levels in *β-cat* ^*S-/*-^ kidneys (1.01 versus 0.48, p = 0.025) (M-O) In situ hybridization and qRT-PCR analysis of *Wnt4* demonstrates a reduction in *Wnt4* mRNA levels in E14.5 *β-cat* ^*S-/*-^ kidneys (1.00 versus 0.30, p = 0.0007). (P) QRT-PCR of Wnt9b-independent gene *Eya1* demonstrates no changes in mRNA expression levels in *β-cat* ^*S-/*-^ kidneys. In contrast, Wnt9b-dependent gene *Tafa5* (1.00 versus 0.42, p = 0.003) was significantly decreased in *β-cat* ^*S-/*-^ kidneys (scale bar = 50μm) (rv = renal vesicle, ub = ureteric bud).

Wnt9b signaling controls genes in the condensed mesenchyme which promote nephron formation through the β-catenin mediated canonical Wnt pathway [24]. To determine whether the reduction in *Wnt9b* expression disrupted Wnt9b downstream signaling, we analyzed the expression of known Wnt9b-independent and-dependent targets in *β-cat* ^*S-/*-^ mutants. We first analyzed the expression Wnt9b independent genes, *Pax2*, *Six2*, and *Eya1*, which are all necessary for the proper induction of the nephrogenic progenitors [26]. First, we performed immunofluorescence for Pax2 and Six2 on *WT* and *β-cat* ^*S-/*-^ mutant kidneys at E15.5. The number of Pax2 positive cells surrounding the ureteric epithelium was markedly reduced compared to *WT*, which is consistent with our finding of decreased induced nephrogenic progenitors (Fig. 8A,B). Using qRT-PCR at E14.5, a time point prior to the reduced progenitor population, we demonstrated no significant change in *Pax2* mRNA expression in *β-cat* ^*S-/*-^ mutant kidneys when compared to *WT* (Fig. 8C). Similarly, immunofluorescence analysis of Six2 revealed a modest decrease in the number of Six2 induced nephrogenic cells (8 D, E) while qRT-PCR showed the levels of *Six2* expression was not changed in *β-cat* ^*S-/*-^ kidneys (Fig. 8F). We also analyzed *Eya1*, a third Wnt9b independent gene [27], which demonstrated no significant alterations in expression in *β-cat* ^*S-/*-^ kidneys (Fig. 8P). Taken together this analysis confirms the reduction in the number of nephrogenic progenitor cells in *β-cat* ^*S-/*-^ kidneys but does not affect the expression levels of these Wnt9b-independent genes. Therefore Pax2, Six2, and Eya1 are not likely contributing to the reduction of the induced nephrogenic progenitors.

To determine if the reduction in *Wnt9b* translates to decreased Wnt9b-dependent downstream targets in the nephrogenic progenitors, we analyzed *Cited1*, *Amph*, *Tafa5* and *Wnt4* [24]. First, we performed immunofluorescence for Cited1 and Amph at E15.5. *β-cat* ^*S-/*-^ mutants demonstrated reduced Cited1 and Amph nephrogenic progenitors. The cells that expressed Cited1 and Amph demonstrated decreased protein expression levels when compared to *WT* (Fig. 8G,H,J,K). We confirmed a 38% and 47% reduction in *Cited 1* and *Amph* mRNA respectively at E14.5 in *β-cat* ^*S-/*-^ kidneys using qRT-PCR (Fig. 8F, I). In addition, Wnt9b dependent targets *Wnt4* and *Tafa5* also demonstrated reduced mRNA expression levels (Fig. 8M-P). Our results are consistent with previous reports in *Wnt9b* hypomorph mice^7^ and Wnt9b null mice [24], and strongly support a role for stromally expressed β-catenin in the regulation of *Wnt9b* expression and its downstream signaling to the condensing mesenchyme.

β-catenin is expressed in distinctive intracellular patterns within the capsular, cortical, and medullary renal stroma. However the role of stromally expressed β-catenin in the regulation of kidney development is not described. Ablation of β-catenin in stromal cells results in numerous kidney abnormalities, most notably a reduction in the condensing mesenchyme. *Wnt9b* expression in the ureteric epithelium and Wnt9b-dependent genes in the nephron progenitors were markedly reduced in *β-cat* ^*s-/*-^ mutants. Our findings support a model by which stromally expressed β-catenin regulates the expression of *Wnt9b* and its downstream targets, thereby regulating the induction of nephrogenic progenitors. The molecular mechanisms by which stromal cells control Wnt9b expression in the ureteric epithelium remain to be determined. We propose three possible mechanisms by which stromally expressed β-catenin modulates *Wnt9b* expression in the ureteric epithelium. First, previous studies have shown stromal cells secrete factors that affect gene expression in epithelial cells in other model systems [19], [28]. The localization of β-catenin to the nucleus in the cortical and medullary stroma suggests β-catenin may control the expression of secreted factors that signal to the adjacent ureteric epithelium to control *Wnt9b* expression. These potential secreted factors are the focus of our future studies. Second, stromal-epithelial cell interactions have been shown to regulate the development of other organ systems [16], [17]. Therefore, it is possible that stromal cells directly interact with the ureteric epithelial population in the kidney. We and others have demonstrated that the renal stroma is directly adjacent to the ureteric epithelium and therefore is ideally located to form direct cell-cell interactions with ureteric epithelial cells to affect cellular processes. Third, recent studies have shown that the renal stromal and condensed mesenchyme form direct cell interactions through stromally expressed proto-cadherin Fat4 [7]. Thus, it is possible that stromal cells, via the mesenchyme cell population, modulate Wnt9b expression in epithelial cells. Taken together our studies highlight that the renal stroma communicates with the ureteric epithelium to modulate gene expression and control kidney development.

## Materials and Methods

### Mice strains and genotyping

*Foxd1EGFPCre* mice were crossed with mice containing LoxP sites flanking exons 2 through 6 (*β-cat^Δ2–6/Δ2–6^*) [21] of the β-catenin allele. The *Foxd1-Cre;β-cat* ^*+/*-^ males were then crossed with *β-cat^Δ2–6/Δ2–6^* to generate homozygous β-catenin loss-of-function mutants in the renal stroma (termed *β-cat* ^*S-/*-^). β-catenin gain-of-function mice were generated by crossing *Foxd1EGFPCre* mice with mice containing LoxP site flanking exon 3 of the β-catenin allele (*β-cat^Δ3/Δ3^*). Tail genomic DNA was isolated and PCR was used to detect the mutants using *Foxd1EGFPCre* primers 5’-GCGGCATGGTGCAAGTTGAAT-3’ and 5’-CGTTCACCGGCATCAACGTTT-3’. Primers used to identify the β-catenin mutants are previously described [21]. To validate the spatial pattern of *Foxd1EGFPCre* recombinase activity the *Foxd1EGFPCre* mice were crossed with *Gt(ROSA)26Sor(ROSA)* mice. All animal studies were performed in accordance with animal care and guidelines put forth by the Canadian Council for Animal Care and McMaster’s Animal Research Ethics Board (AREB) (Animal Utilization Protocol #100855) and approved the project described in this manuscript.

### Histology and Immunofluorescence

Whole kidney tissue was fixed in 4% paraformaldehyde for 24 hours at 4°C. Kidneys were paraffin-embedded, sectioned to 5μm, and mounted on Superfrost Plus slides (Thermo Fisher Scientific, Waltham, MA) and incubated overnight at 37°C. Sections were deparaffinized using xylene washes and rehydrated using graded ethanol washes (100%, 95%, 75%, 50%, H_2_0) and stained with hematoxylin and eosin (Sigma, St. Louis, MO). For immunofluorescence, tissue was prepared as described above and antigen retrieval was performed for 5 minutes in 10mM sodium citrate solution pH 6.0 in a pressure cooker, followed by blocking with serum-free protein block (Dako Corporation, Carpinteria, CA). Sections were incubated with primary antibodies to β-catenin (BD Transduction, Lexington, KY; 1:200), Pbx1 (Cell Signaling, Beverly, MA; 1:250 dilution), Pax2 (Covance, Montreal, QC; 1:200 dilution), Six2 (Proteintech Group, Chicago, IL; 1:250), Cited1 (Thermo Scientific, Fremont, CA, 1:200), Foxd1 (Santa Cruz, 1:200), Meis1/2 (Santa Cruz, 1:200), TN-C (AbCam, Cambridge, MA, 1:200), Amph (Proteintech Group, Chicago, IL, 1:250), Ncam (Sigma, St. Louis, MO, 1:250), Jag1 (Santa Cruz, CA, 1:200), Wt-1 (Santa Cruz, CA, 1:200), Cytokeratin (Sigma, St. Louis, MO, 1:200), Aquaporin-3 (Novus Biologicals, Oakville, ON, 1:200), overnight at 4°C. Tissue sections were washed in PBS pH 7.4, incubated with secondary antibodies Alexafluor 488 or 568 (Invitrogen, Carlsbad, CA; 1:1000 dilution) for 1 hour at room temperature and stained with Dapi (Sigma, St. Louis, MO; 1:1000 dilution) for 5 minutes and cover-slipped using Fluoromount (Sigma, St. Louis, MO) and photographed on a Nikon 90i-eclipse upright microscope.

### Analysis of cell proliferation and apoptosis

Cell proliferation was assayed in paraffin-embedded kidney tissue by incorporation of 5-bromo-2-deoxyuridine (Roche Molecular Biochemicals, Mannheim, Germany), as previously described [29]. Pregnant mice received an intraperitoneal injection of BrdU (100 mg/g of body weight) 2 h prior to sacrifice. BrdU-positive cells were identified using an anti-BrdU peroxidase-conjugated antibody (Roche Molecular Biochemicals, Mannheim). Immunoreactivity was visualized using Aminoethyl Carbazole horseradish peroxidase chromogen/substrate solution (Vector, USA). Apoptosis was assessed in paraffin-embedded kidney tissue using the cell death detection kit (Roche Molecular Biochemicals, Mannheim, Germany) and visualized using 3,3’-Diaminobenzidine (DAB) substrate solution (Vector, USA).

### Real-time reverse transcriptase-PCR

Real-time PCR was performed using the Applied Biosystems 7900HT fast RT-PCR system (Applied Biosystems, Burlington, ON). cDNA was generated using first strand cDNA synthesis (Invitrogen Carlsbad, CA) from total RNA. Real-time PCR reaction mix contained 2.5ng of each cDNA sample, SYBR green PCR Master Mix (Applied Biosystems, Burlington, ON) and 300nM of each primer to a total volume of 25 μl. Primers for *Cited1*, *Six2*, *Pax2*, *Wnt9b*, *Amph*, *Tafa5*, *Eya1*, *Ret*, *Wnt11* were designed using the Primer 3 software (http://bioinfo.ut.ee/primer3-0.4.0/) and verified using the UCSC genome bioinformatics website (genome.ucsc.edu). Relative levels of mRNA expression were determined using the 2^(-ΔΔCt)^ method. Individual expression values were normalized by comparison to β-2-microglobin.

### In Situ Hybridization

Frozen blocks were sectioned at a thickness of 20 μm. Non-radioactive In situ hybridization was performed as described [14] using 1.2μg of either *Wnt9b*, *Ret*, or *Wnt11* digoxigenin-labeled (DIG) riboprobes overnight at 68°C. Sections were treated with 2μg/ml RNase for 15 minutes at 37°C and incubated in anti-DIG-AP antibody (1:2000, Roche) at 4°C overnight and incubated with BM purple at room temperature to visualize signals. Sections were fixed in 4% PFA, mounted in glycergel mounting media (Vector, Burlingame, CA). In Situ Hybridization for *Wnt4* was performed using the Affymetrix QuantiGene ViewRNA assay. Briefly, paraffin blocks were sectioned at a thickness of 5 μm, deparaffinized, boiled in pre-treatment solution (Affymetrix, Santa Clara, CA) and digested with proteinase K. Sections were incubated with a custom designed QuantiGene ViewRNA Wnt4 probe for 2 hrs at 40°C. Signal was amplified with Pre-Amp and Amp solutions and then developed Fast-Red Substrate. Slides were counterstained with DAPI, mounted with Fluoromount (Sigma, St. Louis, MO) and photographed on a Nikon 90i-eclipse upright microscope.

### Statistical Analysis

The qRT-PCR, apoptosis, and proliferation data was analyzed using a two-tailed Student’s t-test using GraphPad Prism software, version 5.0c (Graphpad, La Jolla, CA). P < 0.05 indicates statistical significance.

### Ethics Statement

All animal studies were performed in accordance with animal care and institutional guidelines at McMaster University (Animal Utilization Protocol #100855).

## Supporting Information

S1 Fig The Pbx1 antibody targets the capsular, cortical, and medullary stroma. (A-D) Immunofluorescence analysis of Pbx1, Meis1/2, TN-C, and Foxd1 in *WT* kidneys at E15.5. Pbx1 (A) and Meis1/2 (B) mark the capsular (rc), cortical (cs), and medullary storma (ms), whereas TN-C (C) and Foxd1 (D) expression is restricted to the capsular and cortical stroma between the developing nephrons. The insets demonstrate the expression pattern of each marker in the whole kidney (scale bar = 50 μm, a = adrenal gland, cs = cortical storma, ms = medullary stroma, rc = renal capsule).
(TIF)

S2 Fig Generation of mutant mice with stroma specific deletion of β-catenin. (A-R) Pbx1 and β-catenin immunofluorescence demonstrating the loss of β-catenin from stromal cells in *β-cat* ^*S-/*-^ kidneys. (A-F) At E13.5 in *β-cat* ^*S-/*-^ kidneys, β-catenin is not expressed in Pbx1 positive stromal cells. β-catenin expression is maintained in the ureteric epithelium and mesenchyme populations. (G-R) In *β-cat* ^*S-/*-^ kidneys at PN0 β-catenin is not expressed in capsular, cortical or medullary stroma. β-catenin expression persists in the ureteric epithelium and mesenchyme populations. (scale bar = 50 μm, ub = ureteric epithelium, cm = condensing mesenchyme, s = stroma, cs = cortical storma, ms = medullary stroma, rp = renal papilla, rc = renal capsule).
(TIF)

S3 Fig Characterization of cyst origin in β-cat^S-/-^ kidneys. (A-H) Expression analysis of collecting duct markers Aquaporin-3 (A-D) and Cytokeratin (E-H) in *WT* and *β-cat* ^*S-/*-^ kidneys at PN0. All cysts observed in *β-cat* ^*S-/*-^ kidneys originated from the ureteric epithelium. No cysts were found in the tubules or loops of Henle.
(TIF)

S4 Fig Branching morphogenesis and Nephrogenesis are not disrupted in β-cat^S-/-^ kidneys. (A-F) Whole-mount branching analysis using the ureteric epithelium marker cytokeratin at E13.5 (A,D), E15.5 (B,E), and PN0 (C,F). No overt changes were observed between *WT* and *β-cat* ^*S-/*-^ kidneys. (G-L) Immunofluorescence analysis of nephrogenic markers Ncam (G,H), Jag1 (I,J), and Wt-1 (K,L) in *WT* and *β-cat* ^*S-/*-^ kidneys at E15.5. Despite the reduction in condensing mesenchyme, *β-cat* ^*S-/*-^ kidneys undergo nephrogenesis and form mature nephrons (glomerulus in L). (scale bars A-F = 100 μm, G-L = 50 μm) (rv = renal vesicles, ub = ureteric bud, csb = comma-shaped body, cm = condensing mesenchyme, g = glomerulus).
(TIF)
