# Supplementary figures and images for: Stromally Expressed β-Catenin Modulates Wnt9b Signaling in the Ureteric Epithelium
Source: PLoS One. 2015 Mar 24;10(3):e0120347. doi: 10.1371/journal.pone.0120347 (PMC4372213; doi:10.1371/journal.pone.0120347)

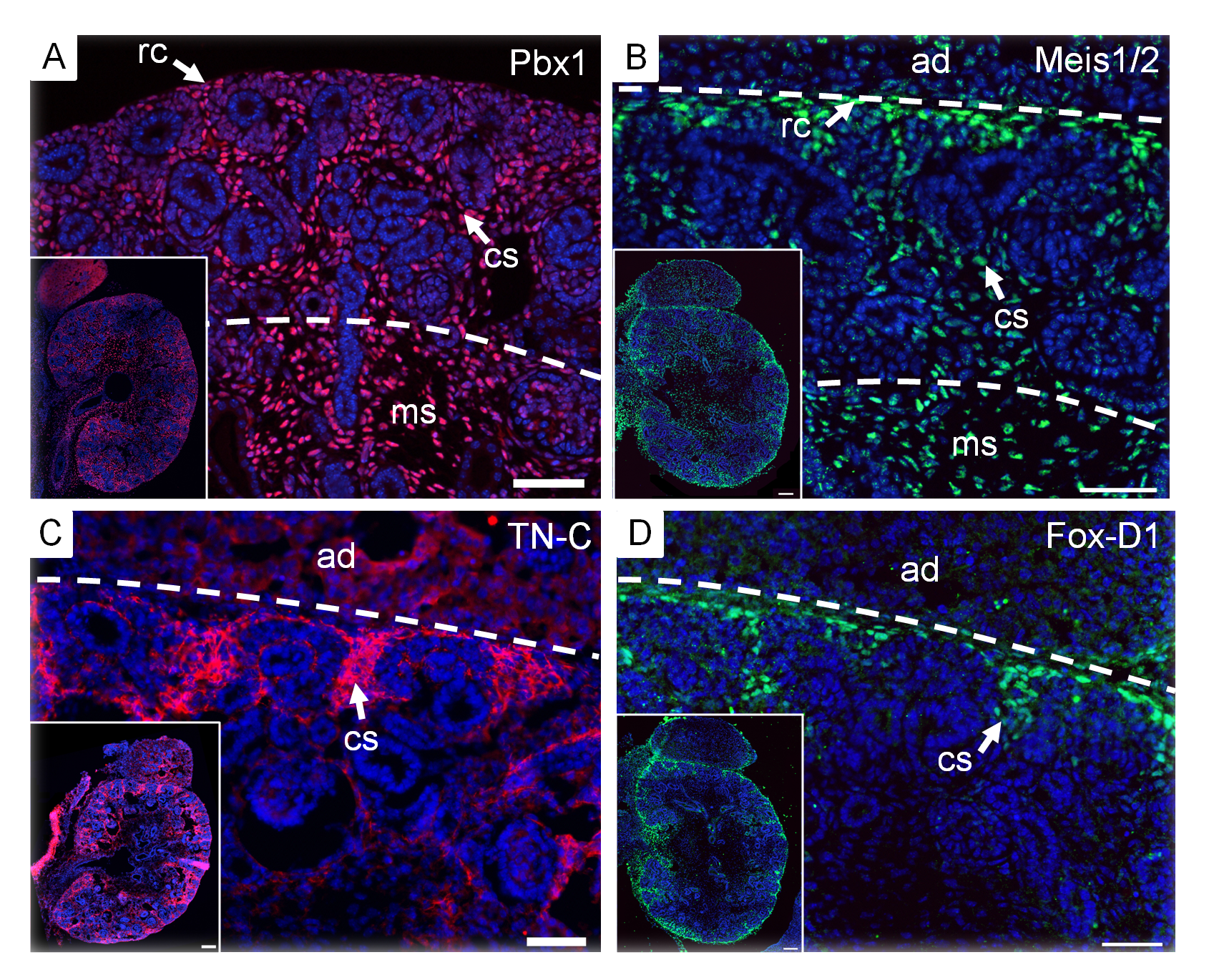

Supplement: S1 Fig — (A-D) Immunofluorescence analysis of Pbx1, Meis1/2, TN-C, and Foxd1 in WT kidneys at E15.5. Pbx1 (A) and Meis1/2 (B) mark the capsular (rc), cortical (cs), and medullary storma (ms), whereas TN-C (C) and Foxd1 (D) expression is restricted to the capsular and cortical stroma between the developing nephrons. The insets demonstrate the expression pattern of each marker in the whole kidney (scale bar = 50 μm, a = adrenal gland, cs = cortical storma, ms = medullary stroma, rc = renal capsule). (TIF) [file pone.0120347.s001.tif]

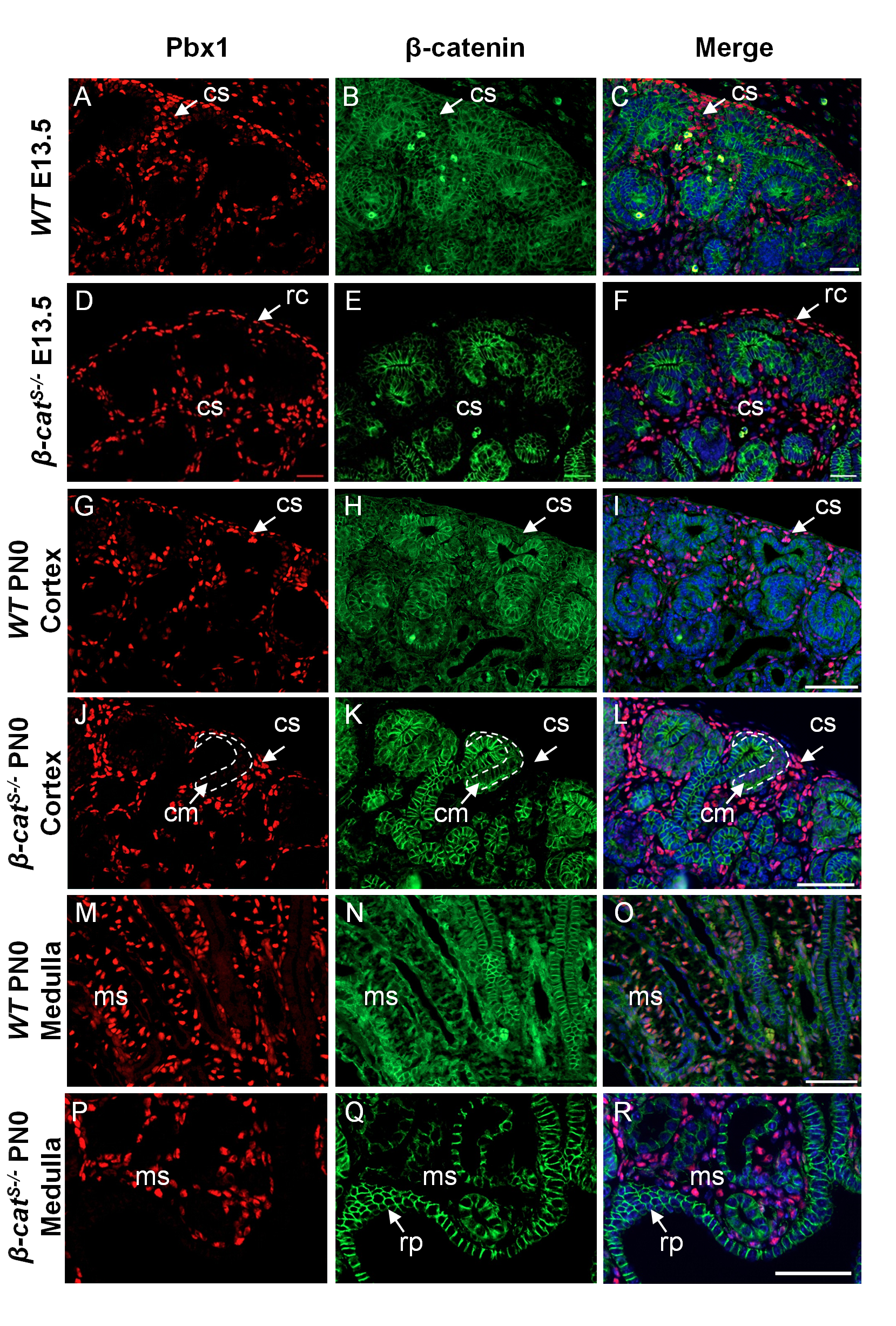

Supplement: S2 Fig — (A-R) Pbx1 and β-catenin immunofluorescence demonstrating the loss of β-catenin from stromal cells in β-cat S-/- kidneys. (A-F) At E13.5 in β-cat S-/- kidneys, β-catenin is not expressed in Pbx1 positive stromal cells. β-catenin expression is maintained in the ureteric epithelium and mesenchyme populations. (G-R) In β-cat S-/- kidneys at PN0 β-catenin is not expressed in capsular, cortical or medullary stroma. β-catenin expression persists in the ureteric epithelium and mesenchyme populations. (scale bar = 50 μm, ub = ureteric epithelium, cm = condensing mesenchyme, s = stroma, cs = cortical storma, ms = medullary stroma, rp = renal papilla, rc = renal capsule). (TIF) [file pone.0120347.s002.tif]

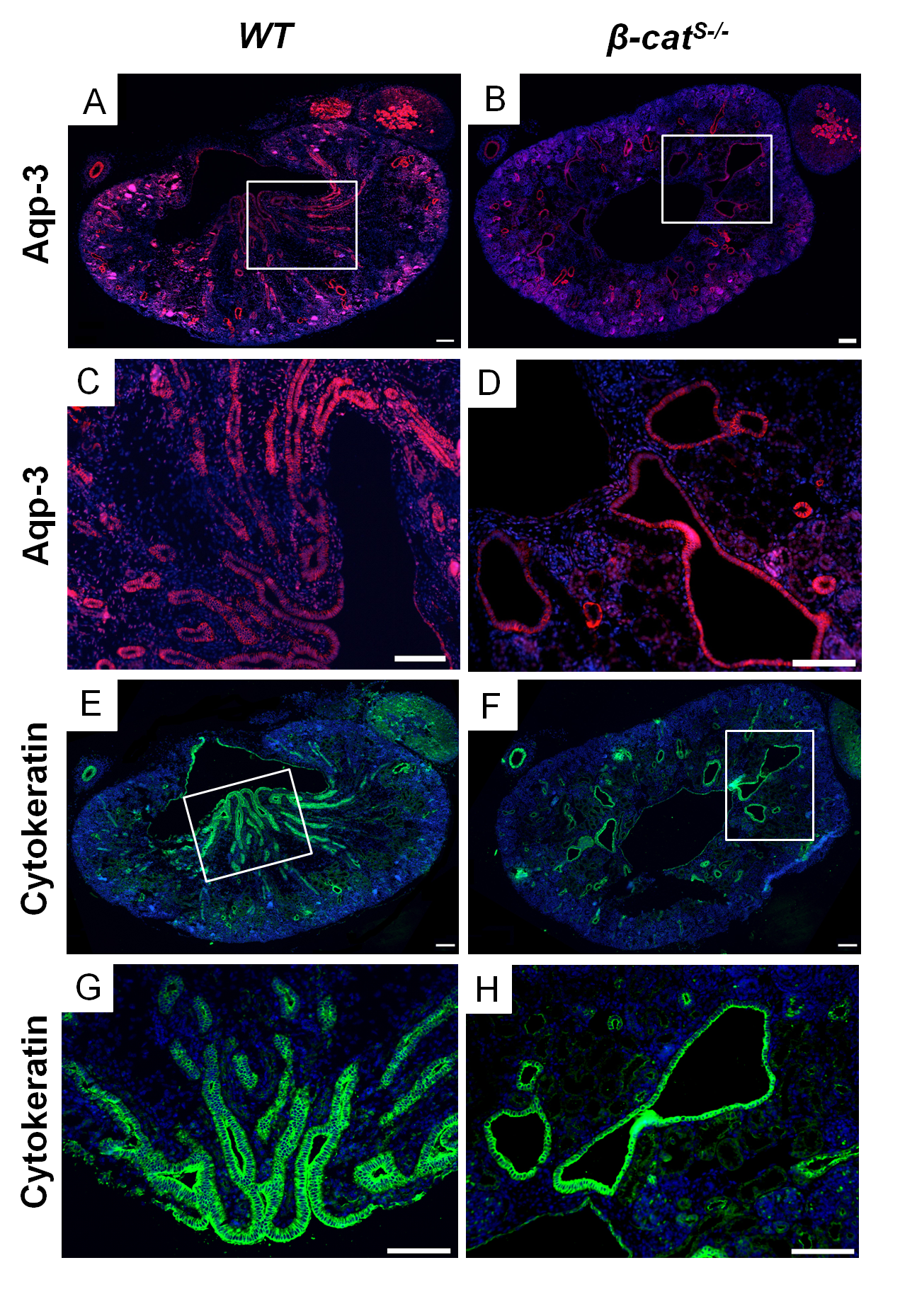

Supplement: S3 Fig — (A-H) Expression analysis of collecting duct markers Aquaporin-3 (A-D) and Cytokeratin (E-H) in WT and β-cat S-/- kidneys at PN0. All cysts observed in β-cat S-/- kidneys originated from the ureteric epithelium. No cysts were found in the tubules or loops of Henle. (TIF) [file pone.0120347.s003.tif]

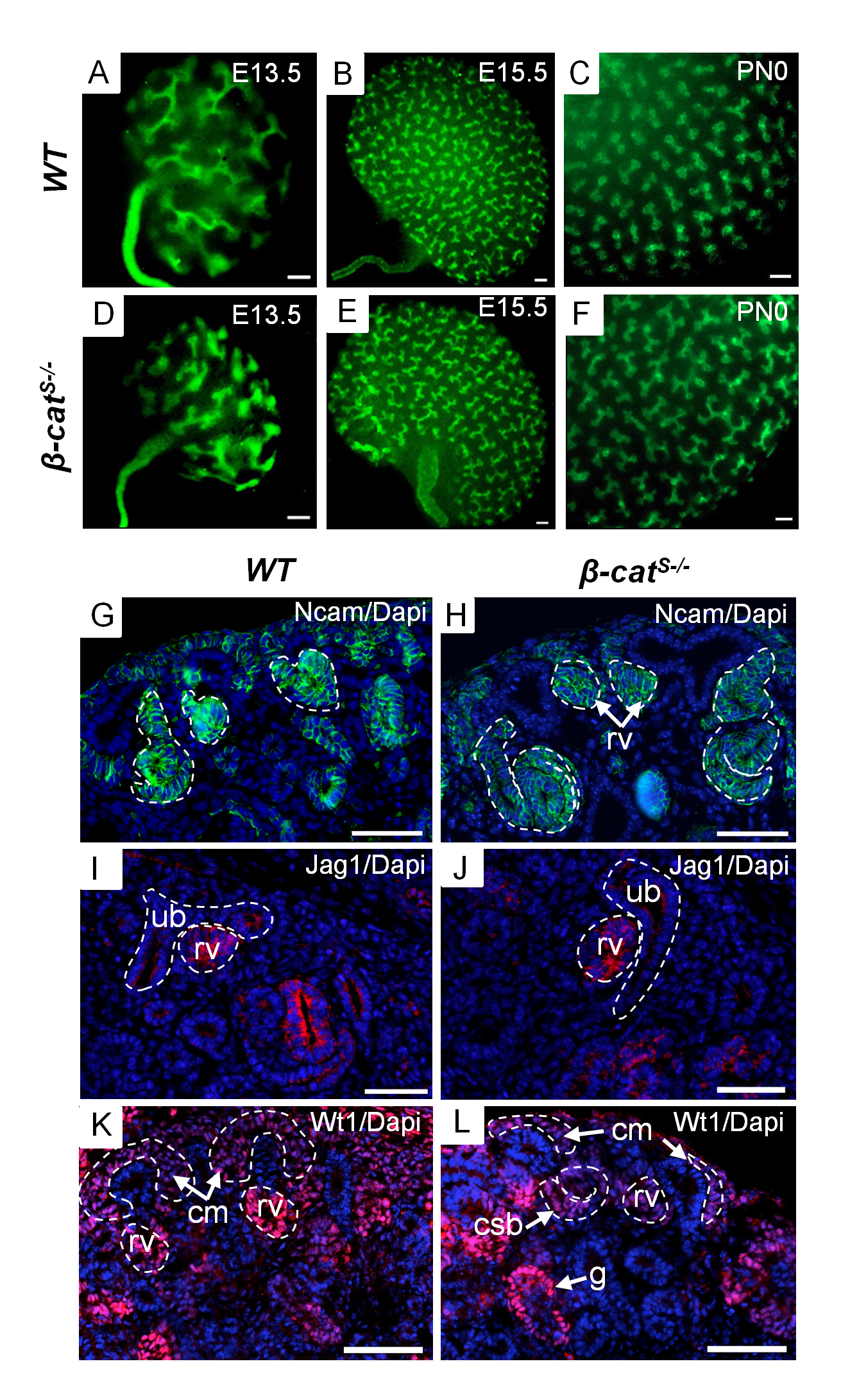

Supplement: S4 Fig — (A-F) Whole-mount branching analysis using the ureteric epithelium marker cytokeratin at E13.5 (A,D), E15.5 (B,E), and PN0 (C,F). No overt changes were observed between WT and β-cat S-/- kidneys. (G-L) Immunofluorescence analysis of nephrogenic markers Ncam (G,H), Jag1 (I,J), and Wt-1 (K,L) in WT and β-cat S-/- kidneys at E15.5. Despite the reduction in condensing mesenchyme, β-cat S-/- kidneys undergo nephrogenesis and form mature nephrons (glomerulus in L). (scale bars A-F = 100 μm, G-L = 50 μm) (rv = renal vesicles, ub = ureteric bud, csb = comma-shaped body, cm = condensing mesenchyme, g = glomerulus). (TIF) [file pone.0120347.s004.tif]
